# A Child’s Concept of Pain: An International Survey of Pediatric Pain Experts

**DOI:** 10.3390/children5010012

**Published:** 2018-01-15

**Authors:** Joshua W. Pate, Julia M. Hush, Mark J. Hancock, G. Lorimer Moseley, David S. Butler, Laura E. Simons, Verity Pacey

**Affiliations:** 1 Faculty of Medicine and Health Sciences, Macquarie University, Sydney, NSW 2109, Australia; julia.hush@mq.edu.au (J.M.H.); mark.hancock@mq.edu.au (M.J.H.); verity.pacey@mq.edu.au (V.P.); 2 Sansom Institute for Health Research, University of South Australia, Adelaide, SA 5001, Australia; Lorimer.Moseley@unisa.edu.au (G.L.M.); david@noigroup.com (D.S.B.); 3 Department of Anesthesiology, Perioperative, and Pain Medicine, Stanford University School of Medicine, Stanford, CA 94305, USA; lesimons@stanford.edu; 4 The Children’s Hospital at Westmead, Westmead, NSW 2145, Australia

**Keywords:** concept of pain, pain, expert survey, children, assessment

## Abstract

A child’s ‘concept of pain’ refers to how they understand what pain actually is, what function pain serves, and what biological processes are thought to underpin it. We aimed to determine pediatric pain experts’ opinions of: (1) the importance and usefulness of assessing a child’s concept of pain in clinical and/or research settings; (2) the usefulness of the content of items within currently published adult-targeted resources for assessing a child’s concept of pain; and (3) important domains of a child’s concept of pain to assess. Forty-nine pediatric pain experts (response rate = 75.4%) completed an online survey. Descriptive statistics and frequency of responses were analyzed. Experts from all included disciplines reported that assessing a child’s concept of pain is important and useful both clinically and in a research setting (>80% reported very or extremely useful for each item). Experts considered that the content of 13 items from currently published adult-targeted resources was useful, but the wording was too complex for children aged 8–12 years. Experts considered that all seven of the proposed domains of a child’s concept of pain was important to assess. The findings can be used to inform the development of an assessment tool for a child’s concept of pain.

## 1. Introduction

Chronic pain is highly prevalent in children and adolescents and has many negative impacts. Reported prevalence rates for different types of pediatric chronic pain range from 11 to 54% [1,2]. An increasing number of children are presenting to hospitals for treatment of chronic pain [3]. Multidisciplinary treatment, including multiple medical and allied health professionals providing a combination of medical management, physical and psychological therapy, is currently considered the most effective and appropriate treatment option [4]. Chronic pain in childhood can predict chronic pain in adulthood [5] and is associated with a negative impact on the quality of life [6], pain-related disability [1], school absence [7], poorer reading scores [8], and emotional distress [9]. Taking into account health service costs ($19.5 billion annually in the USA [10]) and detrimental effects on a child’s mental health, pediatric chronic pain is a significant socioeconomic and health problem.

A person’s ‘concept of pain’ refers to how they understand what pain actually is, what function pain serves, and what biological processes are thought to underpin it [11]. Framed by a biopsychosocial understanding of pain [12] and conceptual change theory [13], each component of a person’s concept of pain is important because each can influence both the pain itself [14] and what one does about it [15]. There is limited literature on how people define pain [16] and their views on its purpose, but there is expanding literature on knowledge of the biological underpinnings of pain [17,18,19,20,21,22,23,24,25]. Adults’ knowledge of pain biology has been assessed in research [24,26,27,28] and clinical practice using the 12-item Neurophysiology of Pain Questionnaire (NPQ) [29], which has been revised (rNPQ) [17] to improve psychometric properties. Importantly, the NPQ was not developed to be used in children and an equivalent tool for that group, who are in a period of rapid cognitive development [30], does not exist. Currently, to our knowledge, no available tool assesses all three components of a person’s concept of pain in either adults or children.

Development of a valid and reliable tool to assess a child’s concept of pain is a critical first step in translating to children the progress made in adult pain research. For example, such a tool is necessary for evaluating educational interventions that target one’s concept of pain—‘explaining pain’ or ‘pain neuroscience education’—interventions that are successful in adults [31] and, anecdotally, widely used in children [32]. Such a tool would allow exploration of whether a child’s concept of pain is associated with current pain and disability due to pain, expectations of outcomes, and engagement with a biopsychosocial model of care, as has been shown in adults [11,31]. Such a tool may also offer predictive value and allow exploration of how successful treatments mediate their effect [33,34,35]. The current study aimed to fill this critical gap for children aged 8–12 years; the age range considered as the first cognitive developmental stage that can be meaningfully interviewed [36,37]. Therefore, as the initial step in developing an assessment tool, the aims of this study were to determine pediatric pain experts’ opinions of: (1) the importance and usefulness of assessing a child’s concept of pain in clinical and/or research settings; (2) the usefulness of the content of items within currently published resources for assessing a child’s concept of pain; and (3) domains of a child’s concept of pain that are important to assess. The opinions of experienced clinicians and researchers working in pediatric pain were sought.

## 2. Materials and Methods

### 2.1. Study Design

We undertook an online survey of pediatric pain experts (Supplementary Materials S1). The survey was designed using Qualtrics software [38] and invitation emails were sent with individualized links to the questionnaire. The survey was available from April to May 2017 and participation was voluntary. Experts gave informed consent to participate in the study and in the online survey. The study was approved by Macquarie University Human Research Ethics Committee (Ref: 5201700229).

### 2.2. Participants

Experts were eligible to be included if they had clinical or research expertise in pediatric pain, as defined by two or more related research publications or two or more years of related clinical experience. The survey was sent to any identified expert deemed eligible by the research team following an extensive search. Experts were identified via tertiary level pediatric pain clinics, those listed as presenters at the 2017 International Symposium on Pediatric Pain (ISPP) conference in Malaysia, from relevant publications, and special interest groups in pediatric pain around the world. Potential participants who had not responded to the initial email were sent reminder emails at 2 weeks, 4 weeks, and 5 weeks; the survey was closed after 6 weeks.

### 2.3. Survey Design

The survey was developed and piloted with two pain clinicians and one pain researcher prior to implementation for this study. This led to improved formatting and to questions that were more concise to reflect ‘concept of pain’ rather than other similarly titled constructs such as ‘self-concept’. The survey collected information regarding the demographics of the responding experts including their role as a researcher and/or clinician, professional discipline, years of experience with pediatric pain and, for clinicians, the number of pediatric pain patients they see in an average week. For this study, a child was defined as being aged 8–12 years.

To achieve the first aim of determining experts’ opinions on the importance and usefulness of assessing a child’s concept of pain in clinical and/or research settings, participants responded to 5-point Likert scales.

To obtain expert opinions on the usefulness of the content of items within currently published resources (the 12-item rNPQ [17] and the 10-item Explain Pain Target Concepts [39]) for assessing a child’s concept of pain, experts were asked to rate (1) the usefulness of the content of each item in assessing a child’s concept of pain and (2) the appropriateness of the wording of each item for children aged 8–12 years. Open-ended questions allowed participants to provide comments and suggest rewording of items.

To identify the domains that are considered by the experts to be important to assess, the team of investigators categorized items from the rNPQ [17] and Explain Pain Target Concepts [39] into seven domains. The seven domains are the following: ‘External influences on pain’, ‘Learning about pain is helpful’, ‘Pain and injury are not closely related’, ‘Pain is about protection’, ‘How pain works’, ‘Things are always changing in your brain and body’, and ‘Pain is a conscious experience’; these are called the Proposed Domains. Participants responded to 5-point Likert scales to rate the importance of assessing each Proposed Domain in children aged 8–12 years. An open-ended question allowed participants to suggest additional domains. Members of the study team who had developed the NPQ (G.L.M.) and the Explain Pain Target Concepts (G.L.M. and D.S.B.) were not involved in collating or analyzing the data.

### 2.4. Data Analysis

The survey response rate was calculated as the number of completed surveys divided by the number of invited experts.

Descriptive statistics were used for the demographics of the experts surveyed and to assess the frequency and distribution of all responses. We evaluated the distribution of the responses for questions using a 5-point Likert rating scale (1 = ’not at all’ through to 5 = ’extremely’). Responses to open-ended questions for comments, justifications, and suggestions were qualitatively synthesized. The median and interquartile range (IQR) number of comments per item was recorded. To identify other domains that may not have been considered, suggestions from experts were noted during data entry. Suggested domains outside the parameters of the survey (i.e., the definition of a child’s concept of pain) were excluded from this analysis.

Because different professionals may hold different perspectives on what is important and useful for children to conceptualize about pain, we compared ratings between professional disciplines, as well as between clinicians and researchers. These comparisons were analyzed by the Kruskal-Wallis one-way analysis of variance to determine any statistically significant differences. For any statistically significant differences between groups, post hoc pairwise comparisons were conducted using a Bonferroni adjustment. Group differences were considered statistically significant at *p* < 0.05.

All values reported were rounded for ease of reading. Data were collated and analyzed using Microsoft Excel 2016 (Microsoft Corp., Redmond, WA, USA, www.microsoft.com) and Statistical Package for the Social Sciences Version 22.0 (IBM Corp., Armonk, NY, USA) [40].

## 3. Results

Sixty-five out of 66 potential participants who were contacted were eligible for the study. Of these, 49 experts completed the online survey and 16 did not respond to emails (75% response rate). The participants included 15 experts who identified as clinical-only experts (31%), 16 as research-only experts (33%), and 18 who identified as a clinical/research expert (37%). The experts represented five professional disciplines and 10 countries. Demographics are outlined in Table 1.

Thirty-two out of 33 experts who identified as clinicians (‘clinical-only’ and ‘clinical/research’ experts) reported that they currently assess children’s concept of pain in clinical practice. Of these, 25 (78%) did this informally based on clinician perceptions, 5 (16%) used their own bespoke questionnaire, and 2 (6%) used the rNPQ [17].

### 3.1. The Importance and Usefulness of Assessing a Child’s Concept of Pain in Clinical and/or Research Settings (1)

**Figure 1:**
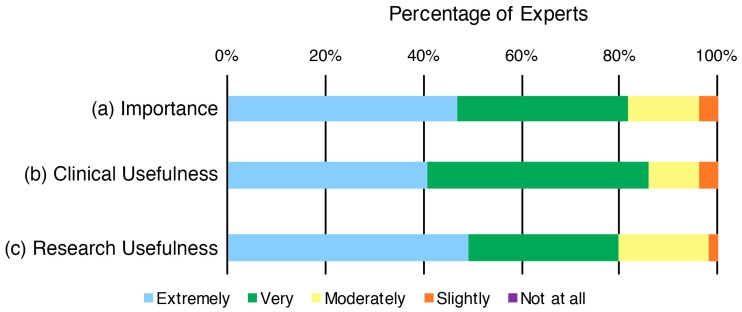
Distribution of pediatric pain experts’ ratings of (a) importance; (b) clinical usefulness; and (c) research usefulness of assessing a child’s concept of pain in children aged 8–12 years.

Most experts considered it as ‘extremely important’ (47%) or ‘very important’ (35%) to assess a child’s concept of pain (Figure 1). Most experts considered it as ‘extremely useful’ (41%) or ‘very useful’ (45%) to assess a child’s concept of pain in a clinical setting (Figure 1) and in a research setting (49% and 31% respectively; Figure 1). No experts stated that assessing a child’s concept of pain would be ‘not at all’ important or useful.

There was no difference in ratings of perceived importance nor clinical usefulness of assessing a child’s concept of pain between the professional disciplines surveyed, which included medicine, psychology, physiotherapy, occupational therapy, and nursing (*p* > 0.2 for both importance and clinical usefulness). Regarding usefulness in a research setting, physiotherapists rated it as more useful than psychologists did (Kruskal-Wallis H-test *p* = 0.015; planned post hoc pairwise Bonferroni-corrected *p* = 0.012; partial eta squared = 0.24) but there were no other differences between professions (*p* > 0.3 for all).

There was no difference between clinical-only, research-only, and clinical/research experts in the perceived importance, clinical usefulness, or research usefulness of assessing a child’s concept of pain (*p* > 0.14 for all).

### 3.2. The Usefulness of the Content of Items within Currently Published Resources for Assessing a Child’s Concept of Pain (2)

**Figure 2:**
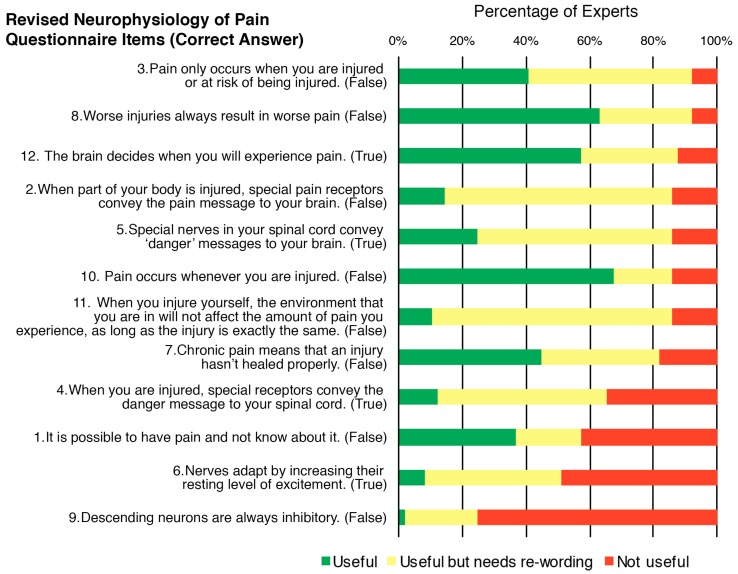
Distribution of pediatric pain experts’ ratings of the usefulness of the content of each item from the revised Neurophysiology of Pain Questionnaire (rNPQ), and the need for rewording, in assessing a child’s concept of pain in children aged 8–12 years.

**Figure 3:**
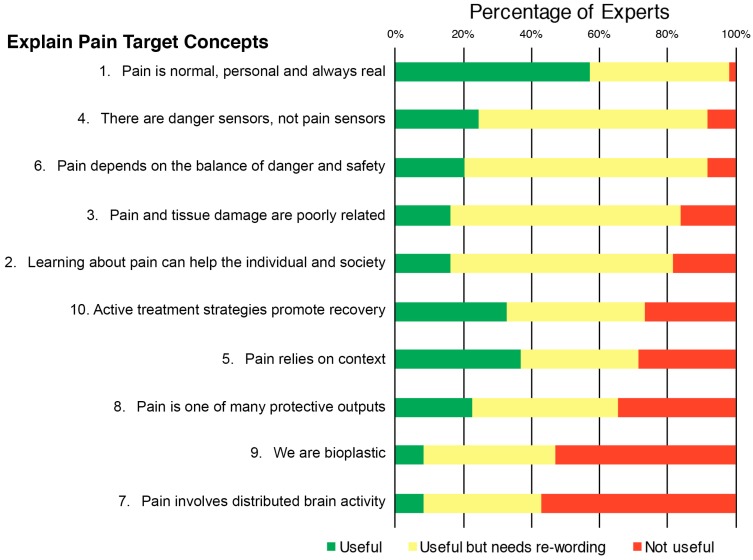
Distribution of pediatric pain experts’ ratings of the usefulness of the content of each item from the Explain Pain Target Concepts, and the need for rewording, in assessing a child’s concept of pain in children aged 8–12 years.

Ratings of the usefulness of the content of each item in assessing a child’s concept of pain ranged from 25 to 92% for the rNPQ items (Figure 2) and 43–98% for Target Concept items (Figure 3). Ratings of the need for rewording of each item ranged from 18 to 76% for rNPQ items and 35–71% for Target Concept items.

In the optional open-ended question regarding comments and suggested rewording for rNPQ items, each item received a median (IQR) of 13 (8–18) comments. Of these, 77% (a median (IQR) of 9 (8–11) comments per item) stated that items were “too complex”. Specific complexities described in these comments included difficulties that children would have with understanding items 1, 2, 3, 7, 8, 9, 10, 11 that have a correct answer of ‘false’ (40%, a median (IQR) of 4 (3–6) comments per item for those eight items) and with items 1, 7, and 11 that contain double negatives (27%, a median (IQR) of 5 (3–6) comments per item for those three items).

In the optional open-ended question regarding comments and suggested rewording for Target Concept items, each item received a median (IQR) of 10 (9–11) comments. Of these, 74% (a median (IQR) of 8 (6–8) comments per item) stated that items were “too complex”. Specific complexities described in these comments for all 10 items included the need to simplify language (39%, a median (IQR) of 4 (3–5) comments per item) and the abstract nature of items (10%, a median (IQR) of 1 (0–2) comment per item).

### 3.3. Domains of a Child’s Concept of Pain That Are Important to Assess (3)

**Figure 4:**
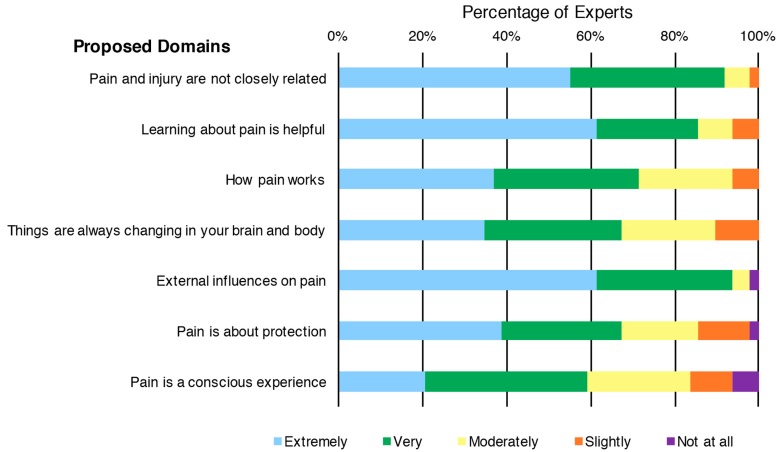
Distribution of pediatric pain experts’ ratings of the importance of the Proposed Domains for a child’s concept of pain, sorted by ratings.

Most experts considered each of the Proposed Domains as ‘extremely important’ or ‘very important’ (59–94% for all) when assessing a child’s concept of pain (Figure 4).

Seven participants made suggestions for additional domains; 10 unique domains being suggested by the group (Supplementary Materials Table S2). However, the authors considered that each of these suggestions was either not relevant to the ‘concept of pain’ definition or they were able to be categorized into one of the original Proposed Domains by the investigators.

For all of the Proposed Domains, there were no between-profession differences (post hoc pairwise Bonferroni-corrected *p* > 0.1 for all).

For six of the seven Proposed Domains, there were no differences between clinical-only, research-only, and clinical/research experts (*p* > 0.1 for all). However, for the ‘Learning about pain is helpful’ domain, clinical-only experts rated it as more important than research-only experts and clinical/research experts (Kruskal-Wallis H-test *p* = 0.01; planned post hoc pairwise Bonferroni-corrected *p* = 0.034 and *p* = 0.017 respectively; partial eta squared = 0.16).

## 4. Discussion

The aims of this study were to determine pediatric pain experts’ opinions of: (1) the importance and usefulness of assessing a child’s concept of pain in clinical and/or research settings; (2) the usefulness of the content of items within currently published resources for assessing a child’s concept of pain; and (3) domains of a child’s concept of pain that are important to assess. The experts reported that it is important and useful to assess a child’s concept of pain. The content of items from the rNPQ and Explain Pain Target Concepts were rated as generally useful to assess a child’s concept of pain, but also rated as too complex for children. All seven of the Proposed Domains were considered important to assess. Ratings were generally similar among the experts from different disciplines, and also between clinical and research experts.

It is critical that the results are interpreted in light of the definition of ‘concept of pain’. Items relating to a more specific and complex understanding of the biological processes thought to underpin the pain aspect of the concept of pain definition were generally rated by experts as less useful to assess. In contrast, items relating to what pain is, the function it serves, and simpler biology items were generally rated as more useful to assess. For example, the content of rNPQ item 9 (“Descending neurons are always inhibitory—False”) and item 6 (“Nerves adapt by increasing their resting level of excitement—True”) were most frequently rated as not useful (Figure 2). Experts’ comments suggested their usefulness related to the complexity of the concepts and the advanced terminology used. This trend was also evident for ratings of the content of Target Concept item 7 (“Pain involves distributed brain activity”) and item 9 (“We are bioplastic”), which were most frequently rated as not useful (57% and 43% ‘not useful’, respectively; Figure 3). Experts in this study reported that many items from currently published resources are not useful for assessing a child’s concept of pain because of their complexity or would need rewording to be used in children. Therefore, it is evident that a way to assess a child’s concept of pain, with sufficient domain coverage and using appropriate language, is required. In addition, any such newly-developed assessment tool would need to be designed to use in clinical practice as well as in research settings given that 97% of surveyed clinicians stated that they currently assess a child’s concept of pain in some form. To ensure the appropriateness for these purposes, the minimal number of questions should be used to reduce burden, and a simple scoring and interpretation method is required [41].

Educational interventions reported in the literature are based on conceptual change strategies [11]; therefore, the individual’s broader concept of pain, rather than knowledge alone, may change in response to these strategies. For example, a recent narrative review stated that most clinical interventions for pediatric pain incorporate education that is relevant to a child’s broader concept of pain [32]. However, an individual’s concept of pain has not yet been assessed in pediatric or adult studies because of the lack of a formal standardized assessment method. Despite this, a 2016 systematic review provided evidence that an educational intervention addressing the concept of pain can improve pain ratings, pain knowledge, disability, pain catastrophization, fear-avoidance, attitudes and behaviors regarding pain, physical movement, and healthcare utilization in adults with chronic musculoskeletal pain disorders [31]. Therefore, the concept of a pain assessment tool could be valuable to evaluate and guide educational interventions and to assess outcomes for a range of children with pain, including those with chronic pain and those with acute post-surgical pain.

Authors suggest that educational strategies for children should be individualized based on needs and capacities [32]. Establishing those needs and capacities currently requires a multidisciplinary assessment that includes aspects of an individual’s concept of pain. However, a validated tool to assess a child’s concept of pain that is designed for children and easily understood would standardize and simplify this process. The concept of a pain tool could be used to efficiently guide educational needs based on a patient’s current concept of pain. The current results suggest that adult tools are not appropriate for children. Therefore, a new pediatric assessment tool encompassing the seven Proposed Domains may provide a helpful starting point for individualized education. In addition, the concept of a pain tool could then also be used by clinicians in re-assessments of patients alongside other validated pain assessment tools to determine the efficacy of specific educational interventions.

Both the clinical and research experts surveyed reported that it is important and useful to assess aspects of biology; however, the complexity of biological information that 8–12-year-old children can conceptualize is not known. The ability of a child to conceptualize pain could relate to cognitive development [42,43], educational level, or a combination of both. It is possible that children are interested in and able to think about how pain works. A survey of 2065 children aged 11 years found overall positive attitudes to science and learning about human biology [44]. Previous studies have shown that children can understand some complex concepts using metaphors [45] or concrete examples [46]. Metaphors have been used in the research literature of pain-related education for adults [47] but not yet in children. Because experts commented on the abstract nature of some items in currently published resources, a consideration of the ‘concrete operations’ Piagetian stage of cognitive development [48] in future research for this 8–12 year age group is necessary. For example, children aged 8–12 years can remember personal events [49], they can link thoughts and feelings in a way that is similar to the way adults do [50], but they are not capable of hypothetical and deductive reasoning [51]. Therefore, further exploration of the complexity of a child’s concept of pain is needed.

### Strengths and Limitations

The multidisciplinary sample of pediatric pain experts from 10 countries was a strength, but it is likely that there are many clinicians who have great expertise but were not identified by our extensive search. Therefore, the experts in this study may not encompass the wider body of expertise. A limitation of our study design was the inability to confirm how the experts interpreted the survey questions. Even though a detailed definition was provided for a child’s concept of pain and clear instructions were given for questions, some experts suggested domains that were outside the definition of a child’s concept of pain. Another potential limitation is that a large proportion of the experts (55%) were from one professional discipline (psychology) (Table 1). This uneven distribution of professional disciplines may have skewed the results, or it may simply reflect that a child’s concept of pain is predominantly being studied by psychologists in the field. Although our exploratory analysis of between-profession differences did not find significant differences with large effect sizes in ratings between professions, the sample was small and was not powered to detect anything but large differences.

To our knowledge, there are no other studies that have reported experts’ opinions on the importance and usefulness of assessing a child’s concept of pain. Directions for future research are to determine children’s understanding of the Proposed Domains and what terminology and language children use to describe their concept of pain. Following this, future research should develop an assessment tool for a child’s concept of pain.

## 5. Conclusions

We found that across multiple professional disciplines, pediatric pain experts agreed that it is highly important and useful to assess a child’s concept of pain. Currently, published resources are seen as too complex for children. Experts rated the seven Proposed Domains derived from adult literature as important to assess regarding a child’s concept of pain.

## Figures and Tables

**Table 1:** Demographics of the pediatric pain experts who participated in the survey.

Characteristic	Experts (*n* = 49)
Clinical-only experts: *n* (%)	15 (31%)
Research-only experts: *n* (%)	16 (33%)
Clinical/research experts: *n* (%)	18 (37%)
Professional discipline: *n* (%)	
*Psychologist*	27 (55%)
*Physiotherapist*	8 (16%)
*Medical*	6 (12%)
*Nurse*	4 (8%)
*Occupational Therapist*	4 (8%)
Years of experience in pediatric pain: *n* (%)	
*2–5 years*	9 (18%)
*6–10 years*	15 (31%)
*11+ years*	25 (51%)
Number of pediatric pain patients per week by clinicians: *n* (%)	
*0*	1 (3%)
*1–5*	9 (27%)
*6–10*	14 (42%)
*11–20*	8 (24%)
*20+*	1 (3%)
Gender: *n* (%)	
*Male*	9 (18%)
*Female*	40 (82%)
Geography: *n* (%)	
*USA*	14 (29%)
*Australia*	13 (26%)
*Canada*	11 (22%)
*Belgium*	3 (6%)
*Denmark*	2 (4%)
*The Netherlands*	2 (4%)
*New Zealand*	1 (2%)
*The Philippines*	1 (2%)
*England*	1 (2%)
*Ireland*	1 (2%)

Note: Characteristics may not sum to exactly 100% due to the effect of rounding.

## Supplementary Materials

The following are available online at www.mdpi.com/2227-9067/5/1/12/s1, Table S1: Online Survey; Table S2: Categorization of domains suggested by experts; Table S3: Data used to create Figure 1; Table S4: Data used to create Figure 2 and Figure 3; Table S5: Data used to create Figure 4.
