# Supplementary material for: A Child’s Concept of Pain: An International Survey of Pediatric Pain Experts
Source: Children (Basel). 2018 Jan 15;5(1):12. doi: 10.3390/children5010012 (PMC5789294; doi:10.3390/children5010012)
Supplement: Supplementary file 1 [file children-05-00012-s001.pdf]

Supplementary Material S1 - Online Survey

Getting started

To ensure you meet the eligibility criteria for this study, please select **all** that apply:

I am a researcher with at least 2 publications related to pediatric pain

I am a clinician with at least 2 years working clinically in pediatric pain

None of the above

Not Eligible

Thank you for expressing interest in this study. Unfortunately you do not fit the inclusion criteria. Please let us know if you would like to be contacted with the results of the study. If you would like any further information, you can contact the team at joshua.pate@mq.edu.au

Participant Information & Consent Form

You are invited to participate in a study of assessing a child's concept of pain.

The aim of this study is to determine expert opinion on content to include in an assessment tool for a child's concept of pain when aged 8-12 years.

The study is being conducted by the following team:

- Joshua Pate (PhD Student, Macquarie University) - joshua.pate@mq.edu.au
- A/Prof Julia Hush (Associate Supervisor, Macquarie University)
- A/Prof Mark Hancock (Associate Supervisor, Macquarie University)
- Prof Lorimer Moseley (University of South Australia)
- Dr David Butler (University of South Australia)
- A/Prof Laura Simons (Stanford University)
- Dr Verity Pacey (Principal Supervisor, Macquarie University)

If you decide to participate, you will be asked to complete a survey online that takes approximately 10-20 minutes. You are free to withdraw at any time without having to give a reason and without consequence.

Individual responses will not be identifiable in any publication of the results, but we will acknowledge those who participate. Only those listed above will have access to the data. The de-identified data will be used to inform future research developing an assessment tool to assess concept of pain in children aged 8-12 years. There is no direct benefit to you, from completing the survey. However, the results from this survey will inform further stages of development of an assessment tool in children, that will become freely available to all clinicians

and researchers. Data from this survey will be published in a relevant journal in this field and presented at national and international conferences as appropriate.

You will be able to access the results of the survey by contacting Joshua Pate on the above email address. The ethical aspects of this study have been approved by the Macquarie University Human Research Ethics Committee (Ethics Approval 5201700229). If you have any complaints or reservations about any ethical aspect of your participation in this research, you may contact the Committee through the Director, Research Ethics & Integrity (telephone (02) 9850 7854; email ethics@mq.edu.au). Any complaint you make will be treated in confidence and investigated, and you will be informed of the outcome.

I understand the information above and I agree to participate in this research.

Yes

No

Purpose of the research

Our team is developing an assessment tool to assess a child's concept of pain, that can be used in clinical practice and research.

The aim of this study is to identify relevant content to include in an assessment tool for a child's concept of pain when aged 8-12 years.

Concept of pain can be defined as how someone understands "what pain actually is, what function it serves, and what biological processes are thought to underpin it" (Moseley & Butler, 2015).

Good news: Your progress will be auto-saved as you go, so you can close your browser window and return to that same computer later (using the link you clicked in your inbox) if you get interrupted.

Demographics

To start with, we would love a few quick details about you...

What best describes your role in pediatric pain? (Select **all** that apply)

Clinician

Researcher

Other:

What is your professional discipline?

Medical

Psychology

Physiotherapy

Nursing

Occupational Therapy

Chiropractic

Other:

How long have you worked in pediatric pain?

Never

<1 year

1-5 years

6-10 years

11+ years

How many pediatric pain patients would you see in an average week?

0

1-5

6-10

11-20

20+

General Pain Questions

Thanks (FirstName). Now we have a few questions based on your experience...

Based on your experience, please list the key constructs (up to 3) you believe are important to assess in children aged 8-12 years about a child's concept of pain?

Do you currently assess children’s concept of pain in clinical practice?

Yes

No

How do you assess children's concept of pain in clinical practice?

Informally based on clinician perceptions

Using the revised Neurophysiology of Pain Questionnaire (rNPQ) Using a modified version of the rNPQ

Using our own questionnaire

Other:

How important do you think it is to assess a child's concept of pain when aged 8-12 years?

Not at all important

Slightly important

Moderately important

Very important

Extremely important

To what extent do you think it would be useful to be able to assess children's concept of pain in **clinical** and/or **research** settings?

	Asssessing concept of pain					
	Not useful at all	Slightly useful	Moderately useful	Very useful	Extremely useful	Comments:
Clinical setting	O	O	O	O	O	
Research setting	O	O	O	O	O	

Assessment tools

Thank you for your input so far! Now comes the exciting part...

The revised Neurophysiology of Pain Questionnaire (rNPQ) assesses knowledge of pain neurophysiology in adults (Catley, O'Connell & Moseley, 2013). As this tool was developed for adults, some of the content may not be appropriate for children aged 8-12 years.

Below are the items in the rNPQ. Please rate to what extent you think the content of each item may be useful for evaluating children's concept of pain. There is a comment box if you would like to provide more details about your answer for each item.

Just a reminder - our assessment tool aims to:

1. *Assess change over time in children's concept of pain.*
2. *Predict clinical outcomes (eg. time missed from school) in children aged 8-12 years with a pain disorder.*
3. *Identify children likely to respond best to a pain education program.*

rNPQ Items (Correct Answer)	Is the **content** of this item useful for assessing children's concept of pain? Yes, include this item as is	No, don't include it	Yes, include this content but it needs rewording into age appropriate language for children aged 8-12 years	Comments/Suggested rewording:
1. It is possible to have pain and not know about it. (F)	○	○	○	
2. When part of your body is injured, special pain receptors convey the pain message to your brain. (F)	○	○	○	
3. Pain only occurs when you are injured or at risk of being injured. (F)	○	○	○	
4. When you are injured, special receptors convey the danger message to your spinal cord. (T)	○	○	○	
5. Special nerves in your spinal cord convey 'danger' messages to your brain. (T)	○	○	○	

	Is the **content** of this item useful for assessing children's concept of pain? Yes, include this item as is	No, don't include it	Yes, include this content but it needs rewording into age appropriate language for children aged 8-12 years	Comments/Suggested rewording:
6. Nerves adapt by increasing their resting level of excitement. (T)	○	○	○	
7. Chronic pain means that an injury hasn't healed properly. (F)	○	○	○	
8. Worse injuries always result in worse pain (F)	○	○	○	
9. Descending neurons are always inhibitory. (F)	○	○	○	
10. Pain occurs whenever you are injured. (F)	○	○	○	
11. When you injure yourself, the environment that you are in will not affect the amount of pain you experience, as long as the injury is exactly the same. (F)	○	○	○	
12. The brain decides when you will experience pain. (T)	○	○	○	

"Target Concepts" have been recently designed to aid the re-conceptualization of pain (Moseley GL, Butler DS (2017) Explain Pain Supercharged, NOIgroup, Adelaide). As this tool was developed for adults, some of the content may not be appropriate for children aged 8-12 years.

Please rate to what extent you think the content of each item may be useful for evaluating children's concept of pain. Examples of related expressions are shown if you hover your mouse over a concept. There is a comment box if you would like to provide more details about your answer for each item.

Just a reminder - our assessment tool aims to:

1. *Assess change over time in children's concept of pain.*
2. *Predict clinical outcomes (eg. time missed from school) in children aged 8-12 years with a pain disorder.*

	Is the **content** of this item useful for assessing children's concept of pain?			
	Yes, include this item as is	No, don't include it	Yes, include this aspect but it needs rewording into age appropriate language for children aged 8-12 years	Comments/Suggested rewording:
**Target Concepts**<br>1. Pain is normal, personal and always real				
2. Learning about pain can help the individual and society				
3. Pain and tissue damage are poorly related				
4. There are danger sensors, not pain sensors				
5. Pain relies on context				
6. Pain depends on the balance of danger and safety				

3. *Identify children likely to respond best to a pain education program.*

	Is the **content** of this item useful for assessing children's concept of pain?			
	Yes, include this item as is	No, don't include it	Yes, include this aspect but it needs rewording into age appropriate language for children aged 8-12 years	Comments/Suggested rewording:
7. Pain involves distributed brain activity				
8. Pain is one of many protective outputs				
9. We are bioplastic				
10. Active treatment strategies promote recovery				

The previous items (from the rNPQ and 10 Target Concepts) have been categorized into subdomains listed below. Please rate how important it is to assess the following subdomains in children aged 8-12 years. If you have any further comments in addition to those you provided above, please add them. There are 3 "Other" rows at the bottom of the table to use if you think of any other subdomains to consider.

	Importance of assessing this **subdomain** in children aged 8-12: Not important at all	Slightly important	Moderately important	Very important	Extremely important	Further comments:
**How pain works** eg. "Receptors send danger messages to your spinal cord then up to your brain where a complex decision about whether or not to output pain".	○	○	○	○	○	
**Pain is a conscious experience** eg. "pain can't be produced without you knowing about it. Many different parts of your brain are involved"	○	○	○	○	○	
**Pain and injury are not closely related** eg. "you can have pain without an injury, and you can have an injury without pain"	○	○	○	○	○	
**External influences on pain** eg. "Pain can be influenced by the things you see, hear, smell, taste and touch, things you say, things you think and believe, things you do, places you go, people in your life and things happening in your body"	○	○	○	○	○	
**Pain is about protection** eg. "Pain depends on the balance of perceived danger and safety."	○	○	○	○	○	
**Things are always changing in your brain and body** eg. "Your brain and body adapt to demands placed on them. If your brain thinks you are constantly under threat for a long time (like in chronic pain), it leads to over-protection"	○	○	○	○	○	
**Learning about pain is helpful** eg. "Understanding pain helps you to set goals and gradually do more"	○	○	○	○	○	
**Other:** (please descibe in "Justification" box)	○	○	○	○	○	
**Other:** (please descibe in "Justification" box)	○	○	○	○	○	
**Other:** (please descibe in "Justification" box)	○	○	○	○	○	

Concluding questions

We really appreciate your input so far. Just one final question...

Are there any other aspects of a child's **concept of pain** that you think should be included in an assessment tool for children aged 8-12 years?

All done!

(Firstname), thanks for completing our survey. Please contact joshua.pate@mq.edu.au if you would like further information.

If you have any other comments, please add them below. (Optional)

Powered by Qualtrics

Supplementary Material Table S2 – Categorization of domains suggested by experts

Suggestions	Thematic categorization of suggestion	Does it fit within the "Concept of Pain" definition?	If 'Yes' to previous column, does it also stand separately from the Proposed Domains?	Result (Include/Exclude)
• *"Rationale for whatever treatment is being implemented"*	Treatment rationale	No	-	Exclude
• *"Items related to child's reality: eg peers, school etc"*	Social situation	No	-	Exclude
• *"Item regarding the role of emotions"*<br>• *"How others respond to your pain impacts your pain experience"*<br>• *"Emotions significantly impact on your experience of pain and its management"*<br>• *"Environment can influence your experience of pain"*	Impact of others/emotions/environment on pain	Yes	No, these fit within the 'External influences on pain' domain	Exclude
• *"Role of genetics and/or psychosocial family factors in pain experience"*	Impact of genetics	Yes	No, it fits within 'External influences on pain'	Exclude
• *"Self-efficacay: You can learn ways to change your experience of pain"*	Learning to change pain experience	Yes	No, this is the 'Learning about pain is helpful' domain	Exclude
• *"Mind-body connection"*	Impact of mind-body connection	Yes	No, it fits within the 'Things are always	Exclude

			changing in your brain and body' domain	
• *"Biopsychosocial model of pain needs to be explained simply"*	Biopsychosocial model	No	-	Exclude

Supplementary Material Table S3 - Data used to create Figure 1.

Answer	Not at all	Slightly	Moderatel y	Very	Extremely
Research Usefulness	0.0	2.0	18.4	30.6	49.0
Clinical Usefulness	0.0	4.1	10.2	44.9	40.8
Importance	0.0	4.1	14.3	34.7	46.9

Supplementary Material Table S4 - Data used to create Figures 2 and 3

Item No.	Is the content of this item useful for assessing children's concept of pain?	Useful	Useful but needs re-wording	Not useful
rNPQ 1	1. It is possible to have pain and not know about it. (F)	36.7	20.4	42.9
rNPQ 2	2. When part of your body is injured, special pain receptors convey the pain message to your brain. (F)	14.3	71.4	14.3
rNPQ 3	3. Pain only occurs when you are injured or at risk of being injured. (F)	40.8	51.0	8.2
rNPQ 4	4. When you are injured, special receptors convey the danger message to your spinal cord. (T)	12.2	53.1	34.7
rNPQ 5	5. Special nerves in your spinal cord convey 'danger' messages to your brain. (T)	24.5	61.2	14.3
rNPQ 6	6. Nerves adapt by increasing their resting level of excitement. (T)	8.2	42.9	49.0
rNPQ 7	7. Chronic pain means that an injury hasn't healed properly. (F)	44.9	36.7	18.4
rNPQ 8	8. Worse injuries always result in worse pain (F)	63.3	28.6	8.2
rNPQ 9	9. Descending neurons are always inhibitory. (F)	2.0	22.4	75.5
rNPQ 10	10. Pain occurs whenever you are injured. (F)	67.3	18.4	14.3
rNPQ 11	11. When you injure yourself, the environment that you are in will not affect the amount of pain you experience, as long as the injury is exactly the same. (F)	10.2	75.5	14.3
rNPQ 12	12. The brain decides when you will experience pain. (T)	57.1	30.6	12.2
TC 1	1. Pain is normal, personal and always real	57.1	40.8	2.0
TC 2	2. Learning about pain can help the individual and society	22.4	42.9	34.7
TC 3	3. Pain and tissue damage are poorly related	24.5	67.3	8.2
TC 4	4. There are danger sensors, not pain sensors	36.7	34.7	28.6
TC 5	5. Pain relies on context	16.3	67.3	16.3
TC 6	6. Pain depends on the balance of danger and safety	32.7	40.8	26.5
TC 7	7. Pain involves distributed brain activity	8.2	34.7	57.1
TC 8	8. Pain is one of many protective outputs	16.3	65.3	18.4
TC 9	9. We are bioplastic	8.2	38.8	53.1
TC 10	10. Active treatment strategies promote recovery	20.4	71.4	8.2

Supplementary Material Table S5 - Data used to create Figure 4.

Importance of assessing this subdomain in children aged 8-12 (% of 49 experts):

Domain	Example	Not at all	Slightly	Moderatel y	Very	Extremely
How pain works	“Receptors send danger messages to your spinal cord then up to your brain where a complex decision about whether or not to output pain”.	0.0	6.1	22.4	34.7	36.7
Pain is a conscious experience	“Pain can’t be produced without you knowing about it. Many different parts of your brain are involved”	6.1	10.2	24.5	38.8	20.4
Pain and injury are not closely related	“You can have pain without an injury, and you can have an injury without pain”	0.0	2.0	6.1	36.7	55.1
External influences on pain	“Pain can be influenced by the things you see, hear, smell, taste and touch, things you say, things you think and believe, things you do, places you go, people in your life and things happening in your body”	2.0	0.0	4.1	32.7	61.2
Pain is about protection	“Pain depends on the balance of perceived danger and safety.”	2.0	12.2	18.4	28.6	38.8
Things are always changing in your brain and body	“Your brain and body adapt to demands placed on them. If your brain thinks you are constantly under threat for a long time (like in chronic pain), it leads to over-protection”	0.0	10.2	22.4	32.7	34.7
Learning about pain is helpful	“Understanding pain helps you to set goals and gradually do more”	0.0	6.1	8.2	24.5	61.2
